# Whole-system approaches to improving the health and wellbeing of healthcare workers: A systematic review

**DOI:** 10.1371/journal.pone.0188418

**Published:** 2017-12-04

**Authors:** Sarah L. Brand, Jo Thompson Coon, Lora E. Fleming, Lauren Carroll, Alison Bethel, Katrina Wyatt

**Affiliations:** 1 European Centre for the Environment and Human Health, University of Exeter Medical School, Knowledge Spa, Royal Cornwall Hospital, Truro, Cornwall, England, United Kingdom; 2 Y Lab Public Service Innovation Lab for Wales, School of Social Sciences, Cardiff University, Cardiff, Wales, United Kingdom; 3 University of Exeter Medical School, South Cloisters, St Lukes Campus, Exeter, Devon, England, United Kingdom; TNO, NETHERLANDS

## Abstract

**Background:**

Healthcare professionals throughout the developed world report higher levels of sickness absence, dissatisfaction, distress, and “burnout” at work than staff in other sectors. There is a growing call for the ‘triple aim’ of healthcare delivery (improving patient experience and outcomes and reducing costs; to include a fourth aim: improving healthcare staff experience of healthcare delivery. A systematic review commissioned by the United Kingdom’s (UK) Department of Health reviewed a large number of international healthy workplace interventions and recommended five whole-system changes to improve healthcare staff health and wellbeing: identification and response to local need, engagement of staff at all levels, and the involvement, visible leadership from, and up-skilling of, management and board-level staff.

**Objectives:**

This systematic review aims to identify whole-system healthy workplace interventions in healthcare settings that incorporate (combinations of) these recommendations and determine whether they improve staff health and wellbeing.

**Methods:**

A comprehensive and systematic search of medical, education, exercise science, and social science databases was undertaken. Studies were included if they reported the results of interventions that included all healthcare staff within a healthcare setting (e.g. whole hospital; whole unit, e.g. ward) in collective activities to improve physical or mental health or promote healthy behaviours.

**Results:**

Eleven studies were identified which incorporated at least one of the whole-system recommendations. Interventions that incorporated recommendations to address local need and engage the whole workforce fell in to four broad types: 1) pre-determined (one-size-fits-all) and no choice of activities (two studies); or 2) pre-determined and some choice of activities (one study); 3) A wide choice of a range of activities and some adaptation to local needs (five studies); or, 3) a participatory approach to creating programmes responsive and adaptive to local staff needs that have extensive choice of activities to participate in (three studies). Only five of the interventions included substantial involvement and engagement of leadership and efforts aimed at up-skilling the leadership of staff to support staff health and wellbeing. Incorporation of more of the recommendations did not appear to be related to effectiveness. The heterogeneity of study designs, populations and outcomes excluded a meta-analysis. All studies were deemed by their authors to be at least partly effective. Two studies reported statistically significant improvement in objectively measured physical health (BMI) and eight in subjective mental health. Six studies reported statistically significant positive changes in subjectively assessed health behaviours.

**Conclusions:**

This systematic review identified 11 studies which incorporate at least one of the Boorman recommendations and provides evidence that whole-system healthy workplace interventions can improve health and wellbeing and promote healthier behaviours in healthcare staff.

## Introduction

Healthcare professionals throughout the developed world have markedly high rates of sickness absence, burnout, and distress compared to other sectors [1–7]. With the added pressure on healthcare systems, and thus on healthcare staff, of rapidly aging populations and burgeoning chronic disease burdens [8], there is increasing interest in improving both the mental and physical health and wellbeing of healthcare professionals [9, 10]. There is a growing call for the ‘triple aim’ (improving patient experience, patient outcomes, and efficiency) to become the ‘quadruple aim’, with the inclusion of improving healthcare staff experience of care delivery [11, 12]. In the United Kingdom (UK) the National Health Service (NHS) in England’s Five Year Forward View [9] identifies NHS staff health and wellbeing as a priority for the NHS.

Sub-optimal health behaviours of healthcare practitioners in the workplace are linked to stress, illness, increased healthcare costs, obesity, high staff turnover, errors, and poor quality healthcare delivery [4, 13]. However, despite concerted policy and research efforts in the last decade designed to support and improve their health and wellbeing (for example [9, 10]), the acute and long term sickness absence of UK healthcare deliverers remains high [14].

Interventions to improve healthcare staff health and wellbeing have primarily focused on supporting or improving individual coping skills rather than affecting the workplace environment such that it promotes healthier behaviours. Whilst personal coping skills mediate the effects of stressors at work on health and wellbeing, i.e. the ability to deal with environmental stressors at a personal level [5, 7, 15], research points to the potential preventative benefits of targeting the workplace at a system-level (including organisational, cultural, social, physical aspects) in creating sustainable and effective health and wellbeing interventions [16].

The Boorman review [17], commissioned by the UK Department of Health to specifically address the health and wellbeing at work of healthcare staff, highlighted the need for whole-system interventions which incorporate input from staff regarding their local needs and contexts and the involvement of management staff at all levels of the organisation. The review proposed five system-level changes for healthcare workplaces to improve staff health and wellbeing: understanding local staff needs, staff engagement at all levels, strong visible leadership, support for health and wellbeing at senior management and board level, and a focus on management capability and capacity to improve staff health and wellbeing. In the United Kingdom, these healthcare workplace improvement plans are supported by the National Institute for Health and Care Excellence (NICE), and are incorporated into the NHS Health and Well-being Improvement Framework [18].

In this systematic review, we sought to identify healthy workplace interventions in health care settings which used elements of this whole system approach and to determine whether they improve the health and wellbeing and promote healthier behaviours in healthcare staff.

## Methods

The systematic review was conducted following the general principles published by the NHS Centre for Reviews and Dissemination (CRD) [19] and is reported in accordance with the PRISMA guidelines [20]. A pre-defined protocol was developed following consultation with topic and methods experts, and is available from the Peninsula Collaboration for Leadership in Applied Health Research and Care (PenCLAHRC) website (http://clahrc-peninsula.nihr.ac.uk/est-projects.php). This study has been reviewed and approved by the Peninsula College of Medicine and Dentistry Research Ethics Committee, now under the auspices of the University of Exeter Medical School Research Ethics Committee.

### Literature search and eligibility criteria

The search strategy was constructed using a mixture of controlled vocabulary terms and free text terms after consultation with topic experts and examination of key papers. The master search strategy is shown in S1 Fig. No language or date restrictions were applied. This search was applied to AMED, CINAHL (via NHS Evidence), Embase, Medline, PsycINFO (all via OVID), SportDISCUS (via EBSCO), the Cochrane Library (via Wiley), Science Citation Index expanded and Social Sciences Citation Index (all via the Web of Knowledge interface). All databases were searched from inception. The main search was run in July 2011, and updated in October 2013 and September 2016. The bibliographies of systematic reviews identified during the screening process and of all papers meeting the inclusion criteria were scrutinised for any additional studies cited. The following websites were searched: UK Department of Health http://www.dh.gov.uk/en/index.htm; UK Department of Work and Pensions http://www.dwp.gov.uk/; US Department of Health and Human services http://www.hhs.gov/; Health Canada http://www.hc-sc.gc.ca/index-eng.php; Australian Government Department of Health and Ageing http://www.health.gov.au/internet/main/publishing.nsf/Content/Home. In addition, the online contents of the American Journal of Health Promotion and International Journal of Workplace Health Management were hand searched for additional articles. These were selected because they were identified as key journals by an expert stakeholder.

### Inclusion criteria

Studies were included if they reported interventions which were targeted at all staff within a healthcare setting (for example a whole hospital, health centre, or unit), were predominantly delivered as group rather than individual activities, and measured the impact on health behaviours or psychological wellbeing in healthcare professionals (outcomes chosen a-priori). Studies in which the intervention was solely aimed at a subgroup of the population (e.g. those with high cholesterol or smokers) were excluded.

Randomised controlled trials (RCT), before and after studies (with or without control), case control, cohort studies and survey designs were included.

### Study identification

Inclusion and exclusion criteria were applied to all titles and abstracts by one reviewer (JP, SLB, AB or JTC) and double screened by a second (JP, SLB, AB, KW, LC, or JTC). Duplicates were identified, checked, and excluded. Discrepancies were resolved by discussion with a third reviewer (LF or KW) where necessary. The full text of potentially relevant articles was retrieved and screened independently by four reviewers (four of: JP, SLB, KW, LC, and JTC); discrepancies were resolved by discussion with a third reviewer (as appropriate, one of: AB, LF or KW).

### Data extraction and quality assessment

A data extraction and quality assessment tool was developed and piloted for suitability on four papers by SLB and KW. Data extraction and quality assessment were undertaken by SLB and checked by KW; any disagreement was resolved through discussion.

Assessment of study quality was carried out using the Newcastle-Ottawa Scale (NOS) for assessing the quality of nonrandomised studies in meta-analyses [21] and the EPOC guidance for randomized controlled trials, controlled before and after studies and interrupted time series (Cochrane Effective Practice and Organisation of Care Review Group [22]).

The following data were extracted from each eligible article (S2 Fig): study design; geographic location of study (country); numbers eligible to participate, numbers participating, loss to follow up; summary characteristics of the study population; details of the intervention; whether the intervention was designed to address a local need; whether and which stakeholders were involved in the development and implementation; whether senior management were involved and in what way, including whether there was visible leadership from or upskilling of management staff; treatment of any control group; duration of follow-up; primary and secondary outcomes, outcome measures and intervention effects. Details on whether the intervention was going to be continued at the site after the initial evaluation were also looked for.

### Data analysis

As the studies as well as the workplace health and wellbeing interventions reviewed were heterogeneous in their design, implementation and outcomes, an overall meta-analysis was not appropriate, rather we aimed to describe the nature of the interventions, whether they engaged staff, and the outcomes. Study and intervention details were put in to tables, with columns for the whole-system recommendations and rows for description of the study/intervention. This supported us to identify patterns in relation to whether and how the studied intervention aimed to 1) engage staff at all levels of organisation in activities and be responsive to local need and context (relating to whole-system recommendations 1 & 2 [17]) and 2) engage, involve and up-skill leadership staff (whole-system recommendations 3, 4, & 5 [17]).

We provide a narrative review of overall patterns of whether and how we believe the interventions in the included studies take a whole-system approach as described in the Boorman recommendations [17], commenting on whether and how the groups of interventions improved the health and/or wellbeing and/or increased health behaviours of healthcare staff.

## Results

### Identified studies

**Fig 1:**
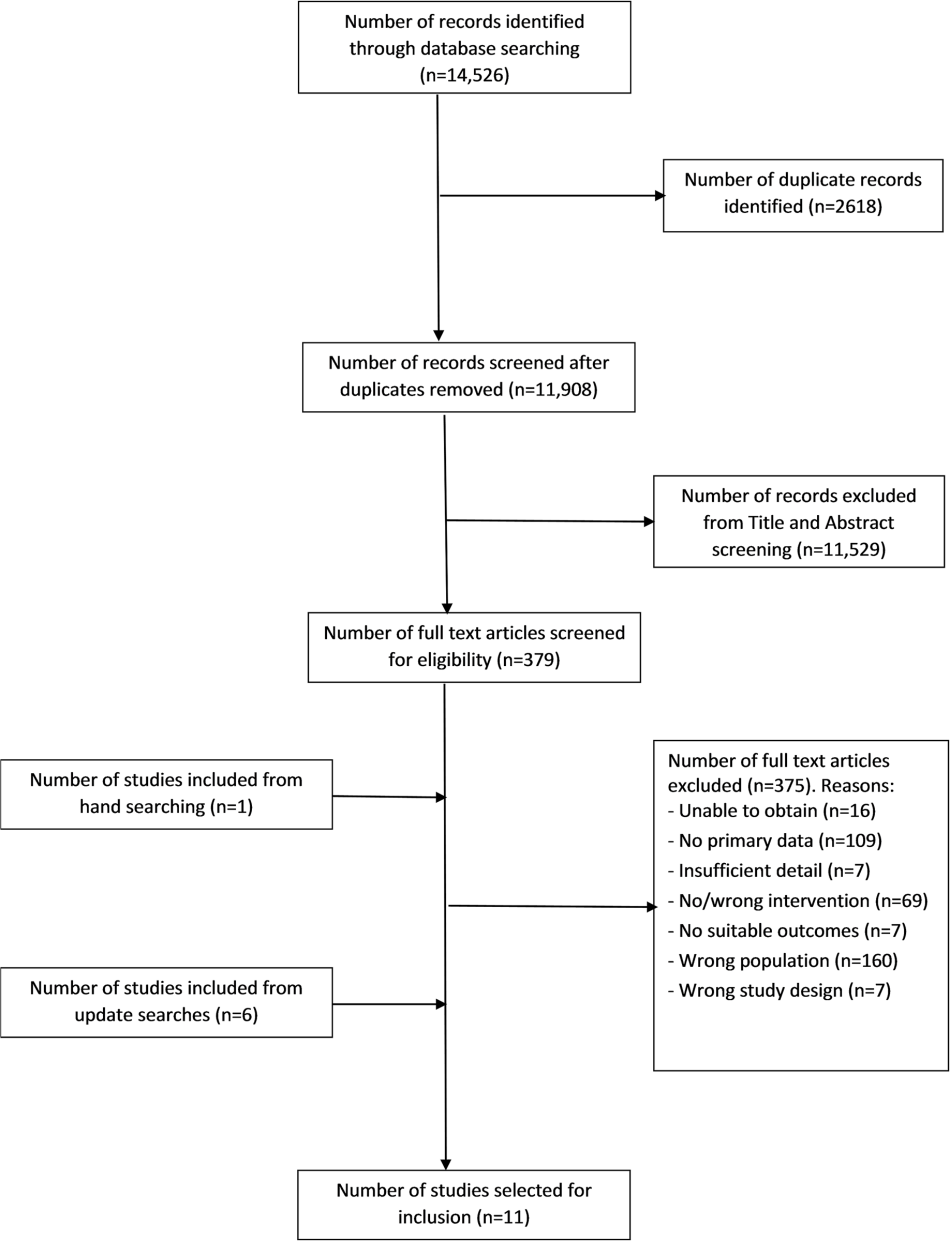
PRISMA flow diagram.

While the original searches retrieved a total of 14,526 records, the review process identified eleven studies to be included (Fig 1). After removing duplicates, 11,908 unique records were downloaded into the reference manager software Endnote to form the master library. The full texts of 379 papers were retrieved for closer examination. Three hundred and seventy five papers were excluded (Fig 1). Update searches (Oct 2013 and Sept 2016) identified a further 7 studies (6 from update searches and 1 from hand searching). A total of eleven studies [6, 23–32] were included, and are summarised in Tables 1–3.

**Table 1:** Summary of design and quality of studies in the systematic review.

**Randomised Controlled Trials**										
**Study**	**Random allocation**	**Treatment allocation concealment**	**Baseline measurement**	**Reliability of outcome measure/s**	**Blinding**		**Adequacy of follow-up (>80%)**		**Protection against contamination**	
Lemon, 2010 [28], USA	Randomised from matched pairs. Method not stated.	None	Completed	Partial	None		Adequate (20% lost to follow-up)		Cluster-randomised	
Sorensen, 1999 [23], USA	Completed. Method not stated.	None	Completed	Insufficient	None		Not reported (baseline and follow-up questionnaires not linked by individual)		Cluster-randomised.	
Sun, 2014 [32] China	Stratified site randomisation	None	Completed	Sufficient	None		Inadequate (50% lost to follow-up)		Cluster-randomised	
Uchiyama, 2013 [29], Japan	Completed. Method not stated.	None	Completed	Sufficient	None		Adequate (20% lost to follow-up)		Cluster-randomised	
**Controlled before-after studies**										
**Study**	**Second site control**	**Treatment allocation concealment**	**Baseline measurement**	**Reliability of outcome measure/s**	**Blinding**		**Adequacy of follow-up (>80)**		**Protection against contamination**	
McElligot, 2010 [26], USA	Convenience sample: Experimental units previously scheduled to programme.	None. Not possible.	Completed	Sufficient	None		Inadequate (>30% lost to follow-up)		Cluster-randomised	
**Before-after studies (no control)**										
**Study**	**Baseline measurement**	**Matching of samples if not same people**	**Reliability of outcome measure/s**	**Adequacy of follow-up (>80)**						
Blake, 2013 [27], UK	Completed	Non-matched samples	Partial	Inadequate (22% lost to follow-up)						
Dobie, 2016 [30], Australia	Completed	n/a	Sufficient	Adequate (none lost to follow-up)						
Hess, 2011 [25], Australia	Completed	n/a	Partial	Inadequate (33% lost to follow-up)						
Petterson, 1998 [6], Sweden	Completed	n/a	Partial	Inadequate (25% lost to follow-up)						
**Survey Studies (no control)**										
**Study**	**Baseline measurement**	**Pre- and post- measures**	**Reliability of outcome measure/s**	**Adequacy of follow-up (>80)**						
Jasperson, 2010 [24], USA	None	No pre-, only 3 months post-events	Low	n/a						
**Cohort study**										
**Study**	**Baseline measurement**	**Representativeness of exposed cohort**	**Selection of non-exposed cohort**	**Ascertainment of exposure**		**Comparability of cohorts**		**Assessment of outcome**		**Length of follow-up**
Wieneke, 2016 [31], USA	None	Somewhat representative	Drawn from same community as exposed cohort	Written self-report		Study controls for any additional factor		Self-report (insufficient reliability of outcome measure)		No follow-up

**Table 2:** Design, outcomes measures, analysis and results of the eleven included studies.

Study	Study design	Outcome Measure/s	Duration of follow-up from baseline (weeks)	Analysis	Results	Results Summary		
						Statistically significant change in physical health	Statistically significant change in mental health and wellbeing	Statistically significant change in health behaviour
Blake, 2013 [27], UK	Before-after study (no control)	Employee questionnaire survey (self-report): *Physical activity* (modified International Physical Activity Questionnaire) Job satisfaction *Perceived general health and mood* (GHQ-12) Sickness absence *Weight*: BMI Perceived work performance	260	Non-matched samples were comparable at baseline and follow-up. Cramer’s V, ANOVA, partial eta squared.	*Physical activity*: Significantly more respondents considered themselves either very or fairly ‘active at work’ at follow-up than at baseline (69.7% versus 53.9%; Cramer’s V = 0.18, p < .001). Significantly more respondents at follow- up reported actively travelling (walking or cycling) to the workplace in the previous seven days (37.6%) than at baseline (30.7%) (φ = -0.07, p < .001). Significantly more participants at follow-up than at baseline reported having walked or cycled for at least 10 minutes in the previous seven days (70.1% versus 65.1%; φ = -0.05, p = .007). Participants who met the recommended level of physical activity were labelled ‘active’, and those who did not as ‘less active’. Significant improvement observed in the proportion of active versus less active from baseline to follow-up (56.4% versus 60.5%; φ = 0.14, p < .001). Respondents at follow-up engaged in significantly more incidental physical activities than respondents at baseline (F(1,2287) = 56.5, p < .001, partial η2 = 0.02). Significantly less time sitting at follow-up (F(1,2149) = 4.9, p < .001, partial η2 = .14) and more time moving about (F(1, 2149 = 9.8, p < .001, partial η2 = 0.17) versus baseline. *Job satisfaction*: Significantly more respondents at follow-up reported being satisfied with their job (F(1,2529 = 11.0, p < .001, partial η2 = 0.004) and feeling committed to working for the trust (F(1,2301) = 5.7, p = .02, partial η2 = 0.002) than respondents at baseline. *General health and mood*: Non-significant trend of lower mood reported at follow-up (8.9%) compared with baseline (12.1%; (φ = 0.01). Overall no significant difference in self-reported general health between baseline and follow-up (Cramer’s V = 0.06, p = .12). Non-significant trends in higher proportion of staff reporting seven hours of sleep more than half of the time at follow-up (61.7%) versus baseline (59.6%; Cramer’s V = 0.04, p = .24), and proportion of smokers reducing from baseline (10.5%) to follow-up (8.6%; φ = 0.03, p = .12). *Sickness absence*: Reported sickness absence levels for the previous month significantly reduced from baseline (4.9%) to follow-up (2.6%; Cramer’s V = 0.13, p < .001). *Weight***:** No significant differences in BMI between baseline (M = 25.2, SD = 4.9, range = 11.2–60) and follow-up (M = 25.4, SD = 5.9, range = 11.5–68.4). *Perceived work performance*: No significant difference pre- to post- intervention on satisfaction with work performance.	O	O	O
Dobie, 2016 [30], Australia	Before-after study (no control)	*The Depression Anxiety Stress Scale (DASS*; validated)—clinical rating of self-reported levels of depression, anxiety and stress. Higher scores reflect greater levels of subjective distress. *The Kentucky Inventory of Mindfulness Skills (KIMS*; validated)—rating of each participant’s self-reported competency in four mindfulness skills: observing; describing; acting with awareness; and accepting without judgement. Higher scores reflect greater levels of subjective attainment. *Brief*, *open-ended feedback questionnaire* (Not validated): surveying their attitudes and experiences toward the programme. Included question: “Score out of ten (ten being most benefit) the extent to which you feel you have benefited from practising mindfulness”	8	Small sample size: Wilcoxon signed-rank tests to evaluate differences between DASS and KIMS mean scores observed before and after. Conventional and summative content analysis to qualitatively analyse feedback questionnaires.	*DASS*: Significant decrease in total scores from 24.67 (77th percentile) to 12.22 (45th percentile; Z = −2.31, p = 0.02); a large effect (r = 0.54). DASS subscales, statistically significant reductions in levels of anxiety (Z = −2.26, p = 0.02, r = 0.53) and stress (Z = −2.12, p = 0.03, r = 0.50). Decreases in self-reported levels of depression were approaching significance (Z = −1.90, p = 0.06). *KIMS*: No significant change over time in the KIMS total score (Z = −1.28, p = 0.20). Non-significant increase in participants’ self-reported competency in observing skills (Z = −1.67, p = 0.09). *Brief open-ended feedback questionnaire*: Thematic analysis: participants’ written feedback was generally positive and all participants reported beneficial outcomes. Two thirds reported a positive impact on body sensations and associated thoughts, including comments of feeling more relaxed, focussed and energised. All participants found the programme allowed them to learn more about stress management, with 90% reporting development of practical new ways of coping with workplace and personal stress.		O	
Hess, 2011 [25], Australia	Before-after study (no control)	*Active Australia questionnaire* (validated; self-report): frequency and duration of physical activity in past week *Health-related behaviour* (self-report): smoking status, self-rated health, physical activity at work, self-rated physical activity level, height and weight (BMI calculated), 4 questions from NSW Health Survey on fruit, vegetable, soft drink, and vegetable consumption.	12	Within group pre-post (related samples Wilcoxon signed rank test and McNemar’s test) Inactive versus active participants (Independent samples Mann-Whitney U test) Qualitative process evaluation	Significant improvement before to after intervention in all health behaviours measured, except for minutes spent doing moderate exercise, ‘healthy’ travel to work, smoking frequency, and feeling depressed (* = p-value calculated with related samples Wilcoxon signed rank test; # = p-value calculated with McNemar’s test): *Active Australia questionnaire*: Physical activity: Number of times spent walking 10 min or more (before: 5 (3–10); after: 7 (5–14); p<0.001*); Minutes spent walking past week (before:125 (60–240); after: 200 (120–345) p<0.001*); Minutes spent doing moderate PA last week (before: 0 (0–90); after: 40 (0–120) p = 0.01*); Minutes spent doing vigorous PA last week (before: 30 (0–120); after: 85 (0–180); p<0.001*); Physical activity ≥150 min per week (before: 71.7%; after: 88.5% p<0.001#) *Health-related behaviour*: Diet: 2 or more serves of fruit / day (before: 57.1%; after: 81.8%; p<0.001#); 5 or more serves of vegetables/ day (before: 10.1%; after: 32.8%; p<0.001#); Breakfast on 7 days / week (before: 56.3%; after: 72.9%; p<0.001#); 1 or more cup of soft drink, cordial or sports drink / day (before: 49.0%; after: 58.3%; p = 0.008#); 4 or more cups of water consumed the previous day (before: 61.2%; after: 80.4%; p<0.001#); Currently on a diet (before: 21.2%; after: 29.0%; p = 0.007#). Mental health: Feeling stressed all the time or most of the time (before: 29.4%; after: 19.6%; p = 0.003#); Feeling depressed all the time or most of the time (before: 6.3%; after: 2.9%; p = 0.08#)		O	O
Jasperson, 2010 [24], USA	Survey study (no control)	*Health questionnaire*: self-reported physical activity and diet (non-validated) Walking event attendance *Post-walking event surveys* (non-validated)	12	None	*Health questionnaire*: After first three months of programme 68% respondents reported better diet and/or more physical activity *Walking event attendance*: 36% (year 1) versus 43% (year 2) of department *Post-walking event surveys*: 96% more motivated to do physical activity; 97% pedometer increased awareness of daily physical activity; 61% activity level same or greater since event; 73% felt department took genuine interest in employees; 76% agreed spirit of teamwork and cooperation in work unit; 74% agreed event promoted staff satisfaction		O	O
Lemon, 2010 [28], USA	Randomised-Controlled Trial	Change in BMI. Body mass index (BMI) was calculated from measured weight and height. Weight measurement was taken on digital scales and rounded to the nearest 2/10 of a pound. Heights were measured to the nearest l/8inch using portable stadiometers. The average BMI across baseline 1 and baseline 2 assessments was used in this analysis. Fruit and vegetable and fat consumption. Fruit and vegetable and saturated fat consumption were measured by the Block rapid food screener, a brief food frequency type measure that assessed commonly eaten foods: 10 items summarised as servings of fruits and vegetables per day. The fat screener consists of 17 items summarised as percentage of total calories from saturated fat. Physical activity. Self-administered long-form of the International Physical Activity Questionnaire (IPAQ), developed by the World Health Organization, with demonstrated reasonable psychometric properties for assessing population levels of self-reported physical activity. Vigorous, moderate, and walking activity in 4 domains, work, household, free time, and transportation, were assessed. Perceived organizational commitment to employee health. 4- item subscale of the worksite health climate survey (WHC), which demonstrated strong internal reliability (a = .88). Respondents rated each item on a 5-point scale. Perceived co-worker normative behaviours. Modified versions of the WHC subscales for health norms measured employee perceptions of eating and physical activity behaviours of co-workers. Individual items were selected and adapted to focus on at-work behaviours. Four items asked about co-workers’ physical activity behaviours at work, and 5 asked about co-workers’ eating habits at work. Seven response categories (almost none to almost all) estimated the proportion of co-workers who practice specific behaviours. Negative items were reverse coded, with higher scores corresponding to healthier behaviours. Psychometric testing of each scale indicated very good internal consistency (a = .78, healthy eating; a = .74, physical activity)	102	Multivariable linear regression models for survey data to assess associations of demographic and job characteristics with the 3 worksite perceptions scales and relationships of the 3 worksite perceptions scales with BMI, fruit and vegetable consumption, saturated fat consumption, and physical activity, controlling for demographic and job characteristics.	Perception of stronger organizational commitment to employee health was associated with lower BMI (B = 0.73, p = 0.03). Higher perception of co-worker normative healthy eating behaviours was associated with greater fruit and vegetable consumption and less fat consumption (B = .33 p < .001). Higher perception of co-worker normative physical-activity behaviours was associated with greater total physical activity (18.2%, p = 0.003). Participation dose-response effect: The more intervention activities people participated in the greater the reduction in BMI: When intervention exposure was used as the independent variable BMI decreased for each unit increase in intervention participation at 24 months (p = 0.006).	O		O
McElligot, 2010 [26], USA	Controlled before-after study	*Health Promoting Lifestyles Promotion (HPLP) II*: (self-report; validated): 52 item Likert scale: 6 subscales—nutrition, stress management, spiritual growth, health responsibility, physical activity and interpersonal relations. High score indicated good health-promotion behaviours, low score indicated poor behaviours. Cronbach’s alpha = 0.93. Six subscales ranged from 0.87 to 0.66.	12 (+2 month response window)	Multivariate ANOVA: pre-post and treatment-versus-control analyses.	Experimental group showed significantly greater increase in overall *HPLP II* score from pre- to post- (F = 15.4, p<0.000) and in 3 of 6 HPLP II subscales from pre- to post-: stress management (F = 17.3, p<0.000), spiritual growth (F = 9.75, p<0.002), and nutrition (F = 10.97, p<0.000).		O	O
Petterson, 1998 [6], Sweden	Before-after study (no control)	Based on results from overall factor analysis, six indices of perceived work quality (competence and skills development, job demands, work pressure, optimal workload, organizational climate and goal clarity), three indices of supporting resources (social climate, job control and coping) and two health indices (psychosomatic symptoms and exhaustion) were measured at baseline and used as outcome measures to evaluate effects of the intervention program. In general, scales used have high internal consistency. Job demands and work pressure had lower internal consistency, questioning their ability to measure unitary dimensions.	52	ANOVA pre- versus post- and high- versus low- activity uptake departments. Based on activity ratings, departments were separated into two groups, one highly active (n = 20) and one less active (n = 17) in the change process. The groups were compared regarding measures of work quality, supporting resources, and health.	Between baseline and follow-up a notice of staff cut-back was announced which was considered a reason for the general deterioration in most measures. All hospital staff were exposed to the same information. Participation dose-response effect: Staff in departments rated as highly active in improvement activities did not deteriorate during the follow-up period compared to a worsening in departments rated as less active in work pressure (active: pre = 14.2, post = 14.2, ns; less active: pre = 14.2, post = 14.5, p<0.01), organizational climate (active: pre = 23.4, post = 23.6, ns; less active: pre = 24.1, post = 24.5, p<0.001) and coping (active: pre = 5.7, post = 5.8, ns; less active: pre = 5.9, post = 6, p<0.05). Amount of activity had no overall effect on staff well-being, perceived social climate, and job control, which decreased for both high and low activity groups.		O	
Sorensen, 1999 [23], USA	Randomised-Controlled Trial	*Employee survey (self-report)*: participation in nutrition-related activities, campaign awareness, and fruit and vegetable consumption *Process tracking system (self-report)*: type and number of interventions, including number of people taking part	104	Pearson product moment correlations calculated to evaluate bivariate relationships between process tracking variable and outcome variables.	Participation dose-response effect: Increase in fruit and vegetable consumption significantly correlated with number of activities per employee (r = 0.55, p<0.05) and percentage of participation in all activities (r = 0.55, p<0.05) Fruit and vegetable intake increased by 0.5 servings (19%) in the worksite-plus-family condition, by 0.2 servings (7%) in the worksite condition. There was no change in the minimal intervention condition			O
Sun, 2014 [32], China	Randomised-Controlled Trial	*Workplace Social Capital (WSC*; validated): assessed by the translated and culturally adapted 8-item measure developed in the Finnish Public Sector study. Cronbach’s alpha coefficients of total scale, horizontal and vertical subscales were 0.90, 0.85 and 0.87, respectively. Following factor analysis of scores the authors divided the eight items into two dimensions: vertical WSC and horizontal WSC. Computed the score of each dimension by summing the scores of all the items in each dimension. The average scores of individual WSC total score, vertical WSC score and horizontal score within each centre were computed to represent the facility-level WSC: Vertical WSC dimension: related to employees’ relations with their employers and supervisors: We can trust our supervisor; Our supervisor treats us with kindness and consideration; Our supervisor shows concern for our rights as an employee. Horizontal WSC dimension: related to bonding and bridging social capital, which involves social contacts, cooperation and trust in relation to co-workers: We have a ‘we are together’ attitude; People feel understood and accepted by each other; People in the work unit cooperate in order to help develop and apply new ideas; Do members of the work unit build on each other’s ideas in order to achieve the best possible outcome?; People keep each other informed about work-related issues in the work unit.	26	Bivariate difference-in-differences (DID) analysis using paired T-test to analyze the facility-level WSC intervention effects. The DID method compares the differences in WSC in pre- and post-intervention periods in the intervention and control groups.	*WSC*: No changes were statistically significant. The facility-level WSC total score, horizontal WSC score and vertical WSC score in the intervention group increased by 1.2, 0.5 and 0.8 points. The same variables hardly changed in the control group. The DID estimators showed that the intervention increased the facility-level WSC total score, horizontal WSC score and vertical WSC score by 1.0, 0.4, and 0.8 points			
Uchiyama, 2013 [29], Japan	Randomised-Controlled Trial	*Mental health status*: Japanese version of the Center for Epidemiologic Studies Depression Scale (CES-D; 20 item, 4 point Likert scale) *Psychosocial work environment*: 3 questionnaires: Job Content Questionnaire (JCQ Japanese version); Effort-reward imbalance questionnaire (short version; ERIQ); Quality work competence (QWC) *Process evaluation*: champions (sub-chief nurses in each work unit) were asked to look back at the whole intervention process of their unit. Researchers’ notes that had been obtained in champion meetings and individual interviews as well as from champions’ task sheets were used. In addition, after the post-intervention survey, champions evaluated the overall interventional process, including descriptive responses	26	Paired t-tests to assess changes in score for each variable in each group. ANCOVA for each variable at post-intervention, controlling for pre-intervention score. Qualitative content analysis for process evaluation	*Mental health status*: No significant intervention effect on mental health status: The change in CES-D score as the primary outcome was not statistically significant (intervention group t = 1.56, p = 0.122; control group t = 1.11, p = 0.268) *Psychosocial work environment*: Some significant effect of intervention on some variables of psychosocial work environment: The intervention group showed a statistically significant increase in the scales of Participatory Management (t = −2.48, p = 0.014), Job Control (t = −2.28, p = 0.024) and Co-worker Support (t = −3.43, p = 0.001), whereas the control group showed a statistically significant decrease in Goals (t = 3.55, p = 0.001). There was also a significant increase in Effort in both groups (intervention group t = −2.08, p = 0.039; control group t = −2.72, p = 0.007). The interaction effect was statistically significant for Goals (F = 8.792, p = 0.003) and Co-worker Support (F = 7.120, p = 0.008). In addition, borderline significance was observed for Job Control (F = 3.840, p = 0.051), even after taking into account the unit variation in scores. Thus although there were significant improvements in psychosocial work environment, these did not improve scores of depressive symptoms *Process evaluation* showed some self-reports of ‘improved work environment’		O	
Wieneke, 2016 [31], USA	Cohort study	A (web-based) survey (validated) was conducted to assess whether the objectives of the wellness champion programme were achieved. The objectives were to increase awareness and participation in healthy living programmes, promote positive health behaviours among employees, and provide a supportive work environment among employees: *Wellbeing*: A validated single-item question was used to assess well-being. *Awareness and participation*: 2 Likert scale items were designed by a health and wellness expert panel to assess awareness and participation in the wellness champions programme. *Self-rated health*: Participants were asked to rate their overall health and wellness on a scale of 0 to 10 (0 being the “worst health and wellness” and 10 being the “best health and wellness”). *The effect of the wellness champions programme on health behaviours*: A team of medical and wellness experts created 2 additional study specific items that asked participants to rate on a 5-point Likert scale, from strongly agree to strongly disagree, their answers to: (1) My co-workers and I support one another in our effort to practice a healthy lifestyle; and (2) My organization provides a supportive environment for its employees to live a healthy lifestyle. Those familiar with the wellness champion program were asked to rank their level of agreement with: Since the introduction of the wellness champion program, I have increased my participation in healthy living programs provided by the organization. The same group of participants was asked: In what ways have you benefited by having the wellness champion program available in your work area?	n/a	The survey items were categorical. Responses to levels of agreement to particular statements (5-point Likert agreement scale) were summarized with percentages. Overall health and wellness (scale from 0 [worst]– 10 [best]) was summarized with percentages (respondents reporting level of 8+) as well as means and standard deviations (SD). Level of agreement and overall health and wellness were compared between program participants versus those not familiar with the program using Wilcoxon rank-sum tests. The survey items specific to the wellness champions program were summarized with percentages among those who reported being aware of the program.	Employees that were familiar and participating in the wellness champion program (N = 666) reported the following benefits of having the wellness champion program available in their work area: 68.5% report an increased awareness of wellness opportunities; 45.2% report having a positive role model for healthy behaviours; 32.6% were guided to new or improved lifestyle habits; 23.3% report an improved work atmosphere; 18.8% were provided with a new trusted resource; 46.9% strongly agreed or agreed that they had increased participation in healthy living programs since the introduction of the wellness champions program Participation dose-response effect: When comparing responses for those who identified themselves as participating in the wellness champion program (N = 666) to those who were not familiar with the wellness champions program but worked in the same work area (N = 675), there were significant differences in responses: Of those participating 82.7% strongly agreed or agreed that the organization provides a supportive environment to live a healthy lifestyle compared to 69.4% of those not familiar with the wellness champions (p < .001); Of those participating in the wellness champions program, 76.8% strongly agreed or agreed that their co-workers support one another in practicing a healthy lifestyle compared to 53.7% of those not familiar with the wellness champions program (p < .001); Those participating rated their overall health and wellness as higher (39.2% with score of 8 or higher on scale of 0–10) as compared to those who were not familiar (33.4%); average rating of 6.9 (SD = 1.5) and 6.6 (SD = 1.7) for the 2 groups, respectively (p = .002) The wellness champions program extended the reach of the onsite wellness center staff, increased engagement, and positively impacted the work environment for many employees Participation in the wellness champions program increased reported overall health and wellness. Participation in wellness champion activities further increased awareness of wellness opportunities, guided employees to new or improved lifestyle habits, and improved the work atmosphere. Participants noted greater support among their colleagues and organization compared to those not familiar with the program		O	

**Table 3:** Summary of interventions in included studies including whether they address aspects of the five whole-system recommendations and their effectiveness at improving healthcare staff health and wellbeing and/or health behaviour change (yes, partial, no).

Study	Intervention	Population /Number approached (no. accepted) /Percentage female	Setting	Engagement of staff at all levels of organisation in activities and responsivity to local need and context (relating to whole-system recommendations 1 & 2, Boorman, 2008)					Engagement, involvement and up-skilling of leadership staff (whole-system recommendations 3, 4, & 5, Boorman, 2008)			Improved the health and/or wellbeing and/or increased health behaviours of healthcare staff (yes O; partial I; no—)
				Developed in response to identified local need	Engagement of all staff in workplace system in group activities to improve health and wellbeing	Choice of intervention activities to participate in	Local staff involved in intervention development / implementation	Adaptive and responsive: ground-up tailoring of activities to local need and context throughout	Strong visible leadership	Support for health and wellbeing at senior management and board level	A focus on management capability and capacity to improve staff health and wellbeing	
Dobie, 2016 [30], Australia	Brief mindfulness based stress reduction (MBSR) programme. It consisted of 15-minutes of group daily guided experiential practice, including five minutes of simple body movements adapted from Thich Nhat Hanh, and 10 minutes of breathing awareness and reflection exercises using scripts adapted from Kabat-Zin, Linehan, Williams, Teasdale, and Thich Nhat Hanh. Sessions ran at the commencement of the morning shift each work day and concluded with an opportunity to debrief. The programme also included three 30-minute education sessions during weeks 2, 4 and 6 designed to increase participants’ understanding of the core components of mindfulness and explore any challenges participants experienced during their practice. MBSR practice focuses on individual coping but the team delivery design of the intervention also enabled whole-system change: Every morning at the beginning of the first shift, the nine staff sat down together for 15 minutes of guided mindfulness practice and five minutes debrief, and there were thirty-minute group education sessions in weeks 2, 4, and 6 to increase participants’ understanding of the core components of mindfulness and explore any challenges participants experienced during their mindfulness practice.	All staff in unit / not stated (9) / f = not stated	Public hospital mental health unit		**O**							**O**
Hess, 2011 [25], USA	Workplace nutrition and physical activity promotion. The intervention ran for a total of 12 weeks. A self-selected group participated in the intervention as only 400 places were offered to the 2900 strong workforce; of those 66% completed the intervention. All participants were provided with a registration pack that included: information leaflet about how the challenge works; pedometer; healthy eating log book; water bottle; sandwich box; ‘Healthy Food Fast’ cookbook; and Measure Up campaign resources. Participants were required to wear a pedometer and record their daily steps for 12 weeks on the 10,000 steps website. Participants were also required to record their daily consumption of fruit, vegetable, water and healthy breakfast in the healthy eating log book during a four-week period, from week five to week eight (for feasibility purposes). Participants’ steps and dietary information were added to produce a team score, which was displayed weekly in the staff canteen. Weekly walks were led by Health Promotion staff during the challenge and were available for all staff at Liverpool Hospital. Other motivational and environmental strategies implemented during the intervention included: posters identifying local walking routes and healthy messages; weekly motivational e-mails; ‘footprints’ directing people to use the stairs; and healthy messages on pay slips. After completion of the challenge, prizes were awarded to the teams who took the most steps and ate the healthiest.	All hospital staff / 2900 (399) / f = 92.8%	Hospital site		**O**							**O**
McElligot, 2010 [26], USA	Promotion of culture of caring and safety. Collaborative Care Model (CCM) program created to promote a culture of caring, focusing on relationships and patient-centred care, fostering and sustaining a healing environment and a culture of safety. The program components were adapted from the Holistic Nursing Handbook and best practice models (Dossey & Keegan, 2009). The didactic content included interactive lectures on the CCM program, American Holistic Nurses Association values, formation of the collaborative care council, and a code of professionalism. The experiential content included completion of the Health Promoting Lifestyles Promotion II tool, option for study participation, and experiences with imagery, appreciative inquiry, and a sharing circle. Aim of the intervention was for participants to be able to: Define the CCM as the professional practice model of the institution; Relate the CCM to the five core values of the AHNA; Participate in the self-assessment of personal health-promotion behaviours through tools and discussion; Demonstrate the use of appreciative inquiry as a method of change; Identify one self-care health-promotion goal and one group health-promotion goal. Activities included: interactive lectures; HPLP II tool completion; self-assessment of personal health-promotion behaviours; discussion; healthy behaviour goal-setting; experience of imagery; appreciative enquiry method for change, sharing circle.	103 registered nurses / 408 (270) / f = 95%	Hospital units	**O**	**O**	**O**						**O**
Blake, 2013 [27], UK	NHS workplace wellness intervention (including: dedicated website; timetable of exercise sessions; staff gym; cycle storage and showers; slimming classes; healthy eating schemes; health campaigns such as wellbeing week, active commuting, and mental health week). Workplace champions were employed to promote the services and facilities. Champions were identified as employees who recognised importance of health and wellbeing and were paid to do this work during their core hours.	All hospital staff / 7065 (1452) / f = 80%	Large NHS organisation		**O**	**O**	**O**					**O**
Sorensen, 1999 [23], USA	Treatwell 5-a-Day for Better Health campaign incorporating three key theoretical constructs: 1) employee involvement, 2) socio-ecological approach targeting intrapersonal, interpersonal, and organisational influences on eating behaviour, 3) the use of adult learning and behaviour change strategies. Intervention included: newsletters; posters; nutrition education hour and 10 session discussion series; multiple themed activities; organisational environment changes, for example point-of-purchase labelling and vending machine signage; and family activities, for example family festivals, health fairs, picnics, and Fit in Five learn-at-home nutrition education programme. The study compared a minimal intervention group (i.e. no activities, public awareness campaign, and one hour of nutrition education), a worksite-only group (i.e. all elements of intervention plus worksite activities), and a worksite-plus-family group (i.e. all elements of intervention plus worksite-plus-family activities, family festivals, and Fit-in-Five at-home education program.	1306 community health centre staff / 1588 (1359) / f = 84%	22 community health centres		**O**	**O**	**O**					**I**
Jasperson, 2010 [24], Sweden	Wellness program developed by two departments at hospital and delivered by a part-time coordinator and 17 champions from departments from a variety of job roles who met monthly. Main activities were three annual team walking competitions in which pedometer steps per day were added up for each team and the progress of each team in miles across a map was presented in a shared area of the hospital. Walking competitions happened yearly and lasted 2 months. Other activities included lectures.	1700 hospital staff / 1700 (year 1 = 610, year 2 = 812) / f = not reported	2 hospital departments	**O**	**O**	**O**	**O**					**I**
Sun, 2014 [32], China	Workplace Social Capital intervention including four activities: Team leadership training activity (one activity): A one-day team building courses for directors (team management and communication skills and practical team leadership experiences). The directors in intervention centers were asked to join and coordinate all non-leadership activities. Non-leadership activities for staff (three activities): Self-organizing voluntarily public services for disadvantaged community residents (each intervention center was asked to self-organize public services for the older adults, the disabled or the poor within their communities); Half-day group psychological consultation (half-day consultations for each center focusing on team communications and stress management); One-day outdoor experiential trainings aiming at improving team coordination and communications.	480 staff / (447) / f = not stated	20 community health centres		**O**	**O**	**O**		**O**	**O**	**O**	—
Lemon, 2010 [28], USA	One of seven projects in the National Heart, Lung, and Blood Institute: Overweight and Obesity Control at Worksites initiative. Employee and leadership advisory committees helped develop site-tailored strategies to promote organisational and social norms related to eating and physical activity in the workplace to improve health behaviours and BMI. The Step Ahead ecological intervention approach targets change at the organization, interpersonal work environment, and individual levels. The intervention was developed using participatory research. Engaged leadership support and assistance during intervention development stage and involved them in development of the intervention. “Top down” approach of first engaging the support of top leadership. Strong leadership support was made clear to cafeteria and facilities middle management and staff members, whose cooperation was needed to implement changes. Employee involvement in intervention planning and development in focus groups. Overall the groups were enthusiastic about the project. Involved in suggesting and discussing potential activities prior to implementation. All staff invited to participate in focus groups at each hospital. Activities/interventions included: organisational leadership, climate, culture, and capacity to promote an environment supportive of weight control; social marketing; walking groups; signs on stairs; walks with the president; nutritional information in café; seasonal farmers market; individual and group challenges.	1983 hospital staff / 1983 (899 accepted, 806 took part) / f = 81%	6 hospitals from one healthcare system	**O**	**O**	**O**	**O**		**O**	**O**	**O**	**I**
Petterson, 1998 [6], Sweden	Inclusive, staff-led intervention ‘process’, departments used own autonomy to choose intervention elements, group goal setting, communication, cooperation, and social relations within each department. The intervention program was initiated by the hospital management with a large questionnaire study of work environment and health of all hospital staff. Each department management and staff were encouraged to engage in the improvement of their own work environment. Survey feedback was a means to get all staff involved in the process by initiating discussions on local work problems, needs for improvements and to stimulate activities to change negative work conditions. Based on survey feedback of results presented as histograms, of its own department values comparable to other departments and hospital mean values, each department had to choose those improvement areas most relevant to its organization, to make goals for workplace improvements and to plan for activities to realize those goals. All staff were encouraged to contribute to the formulation of goals as well as to take part in decided activities. A lot of activities were initiated but there was a great variation across departments with regard to time spent and to choice of activities. Each department had the possibility to apply for financial support for special activities. Next to more common activities such as group discussions on new competence needs, supervision, leadership qualities, information channels, work or meeting routines, flexible working hours, and on organizational goals and visions, separate department programs included study visits to other hospitals, quality circles or cources, debriefing or physical training. Lecturers were invited to talk about issues like work and stress and consultants were engaged to investigate the needs of new competence of the local organization. Most of the departments also arranged social activities. Department programs were primarily expected to facilitate communication and cooperation, to increase staff participation, and also to improve work efficiency and social relations which in turn was supposed to improve perceived work quality, supporting resources, and staff health and well-being.	2617 hospital staff / 4613 (3506) / f = not stated	Hospital departments	**O**	**O**	**O**	**O**	**O**	**O**	**O**		**I**
Uchiyama, 2013 [29], Japan	Participatory intervention for psychosocial work environment: all employees were invited to share good practice and barriers to working, including planning of problems, needs, progress and creating a plan of activities. The intervention was unit based, focused on active employee participation, and based on action planning to improve the work environment. All members in the intervention units were expected to participate in a series of activities designed to improve the work environment. Development: Results of a pre-intervention survey were reported to each unit and used for target identification and prioritization of the targeted psychosocial work environment, and as an index of improvement. In reference to their own unit’s results, all members of the unit were asked to describe their ideal work environment and invited to develop action plans to improve their psychosocial work environment. Comprehensive information on mental health in the workplace and psychosocial work environment as a source of stress was provided to each unit. Champions: Sub-chief nurses in each intervention unit were appointed as champions to facilitate activities within their own units. 30-minute group meetings of champions to share information on good practices and obstacles. 30-minute individual interviews with each champion conducted by the first author to provide advice on facilitating other staff activities in their units. Champions then shared necessary information with staff of their own units. Champions filled out task sheets after every 30-minute group meeting to clarify the problems, needs, and progress of their unit and to help plan execution of the activities. Champions were assigned to list the issues of their own units that needed to be improved and incorporate the opinions of unit members. They identified existing problems, while considering the effectiveness, feasibility, priority, and time cost of improvements. Implementation: Nurses in the intervention group started to improve their psychosocial work environment based on the action plans proposed in the development phase. Suggestions for further improvement and sustaining autonomous activities were discussed during this period.	496 nurses in units / 434 (401) / f = 100%	Hospital units	**O**	**O**	**O**		**O**	**O**	**O**	**O**	**I**
Wieneke, 2016 [31], USA	The wellness champion program was designed to improve the health and wellbeing of employees by extending the reach of the onsite healthy living programs and staff into the worksite to create a supportive work environment for having a healthy lifestyle. A multistep process was utilized to implement a cost-effective wellness champion program across the organization. Workplace wellness champions created workplace wellbeing activities from a range across several domains for their local work area. These activities were intended to impact the culture of health through organisational and peer support for employees. Workplace wellness champions are provided ready-made program resources and given the autonomy to promote programs of personal and work group interest for their local work group, including physical activity, volunteerism, teambuilding, social interaction, stress management, and new experiences such as healthy potlucks, walking or stair-climbing campaigns, and team weight-loss competitions. Wellness champions promote health and wellness opportunities via print, electronic, and in-person communications. The first two worksite wellness champions designed the intervention, resources and the training of the more than 440 now existing workplace wellness champions.	4129 staff with workplace wellness champion in their local work area; (2315) of which 1630 were familiar with the workplace wellness program / f = not stated	One large academic medical centre	**O**	**O**	**O**	**O**	**O**	**O**	**O**	**O**	**O**

### Study characteristics and quality

#### Outcomes

All reviewed studies included self-reported measures of individual health behaviours and health outcomes, and four studies [6, 23, 28, 29] included self-reported measures of the psychosocial workplace environment (see Table 3). Two studies [28, 32] reported Body Mass Index (BMI).

#### Study design and quality

The overall quality of included studies was considered to be poor (Table 1). The main reasons relate to the outcome measures, which were generally low in reliability, variable in validity, and heterogeneous; lack of controls; variable follow-up length; and high attrition rates.

Reliability and validity of outcome measures: Six [6, 23–25, 27, 28, 31] out of the eleven studies had partial or low reliability of outcome measures. Three studies [6, 23, 24] did not use validated outcome measures. Petterson and colleagues [6] used self-report scales based on the findings of a factor analysis. They report that in general these scales have high internal consistency, though two (job demands and work pressure) had lower internal consistency, leading them to question the ability of these scales to measure unitary dimensions. Some studies used self-report measures. Sorenson and colleagues [23] used self-report survey and process tracking measures (including self-reported number of activities taken part in). Jasperson and colleagues [24] constructed and used a health questionnaire that measured self-reported physical activity and diet and a survey measuring self-reported walking event attendance.

Heterogeneity of outcome measures: Three studies [25, 27, 28] used an objective outcome measure of health (BMI). The other nine studies used subjective self-report measures, and no two of them used the same subjective self-report measures.

Study design: In addition (see Table 1) five of the eleven studies lacked a control group [6, 24, 25, 27, 30], two had no follow-up [24, 31], and one did not report follow-up information because baseline and follow-up questionnaires were not linked by person [23]. Follow up periods varied considerably; in six studies [23–26, 29–31] follow-up (or single time-point) data were collected immediately post intervention. In the remaining studies follow-up data were collected at between 3 months [26, 32] and 5 years [27] after the start of the programme. Follow-up rates also varied as the workforce itself changed over the follow-up period.

Attrition rates: Five of the eight studies reporting follow-up had attrition rates higher than 20% (ranging from just over 20% to 50%) [6, 25–27, 32]. One study reported no attrition; two studies reported 20% attrition (Lemon, Uchiyama); four studies [6, 25–27] reported attrition rates varying from 22 to 33%; one study reported 50% attrition, one [23] did not report attrition rates; and two did not have baseline measurements [24, 31].

Participation: Nine studies [6, 23, 24, 26–32] offered the intervention to all hospital/unit/health centre staff; one study [25] offered the intervention to everyone, but operated on a first come first served basis as there were only 400 places available to the 2900 staff; one study [31] offered the intervention to all staff working in a work area that had a workplace wellness champion working in it. Given the nature of the included interventions (i.e. aiming to affect whole-system change), it is hard to estimate overall participation rates other than in the specific activities which were delivered within the intervention programme.

None of the studies described the interventions in sufficient detail to allow replication. One study [30] offered the manual for their brief MBSR intervention upon request.

### Effectiveness of interventions

#### Included studies

All interventions were deemed by their authors to be at least partly effective (Table 2). Two studies reported statistically significant improvement in objectively measured physical health (BMI; [27, 28]) and eight in subjective mental health [6, 24–31]. Six studies reported statistically significant positive changes in subjectively assessed health behaviours [23–28].

Due to the heterogeneity of types of study and measures used, it is difficult to make meaningful comparisons between the studies. We describe the interventions in relation to the degree to which they included the whole-system recommendations for healthy workplace interventions in healthcare settings [17].

#### Included interventions

Interventions varied in terms of whether and how they incorporated the five whole-system recommendations [17] (Table 3) and their overall effectiveness (as reported by the authors of each study) in improving healthcare staff health and wellbeing and/or health behaviour change (Table 3: yes = O; partial = I; no = —).

### Recommendations 1&2: Identifying and responding to local need and engaging staff at all levels

The eleven studies varied considerably in how they tailored their interventions to local need and engaged staff at all levels (Table 3; [17]). Interventions were: 1) pre-determined and fixed from the outset without choice of activity [25, 30]; 2) pre-determined with choice of activity [26]; 3) had choice of a wide range of activities and some adaptivity of the programme, with further activities added in response to take-up [23, 24, 27, 32]; and, 4) adaptive and responsive workplace programmes, taking a participatory approach from the beginning and creating programmes responsive and adaptive to staff needs, in which the implementation process was part of the intervention [6, 28, 29, 31].

#### 1) Pre-determined interventions with no choice of activities

Two studies offered a fixed set of activities, including some element of group activities, to all staff in one workplace. These activities were not created in response to local need, and nor was there choice about which activities to participate in [25, 30].

An eight-week Mentalisation-Based Stress Reduction (MBSR) intervention for staff in a 12 bed mental health inpatient unit (MBSR practice focuses on individual coping but the team delivery design of the intervention also enabled whole-system change; Table 3) resulted in a significant decrease in self-reported psychological distress, including reduced levels of self-reported anxiety [30]. There was no overall increase in the Kentucky Inventory of Mindfulness Skills, suggesting that these changes in distress and anxiety may have resulted from the increased communication and activity-sharing between the work unit [30].

Implementing a pre-determined 12 week intervention to improve physical activity and nutrition behaviours across a hospital site using a team-based approach and peer support (Table 3) resulted in those completing the intervention reporting significantly higher physical activity, fruit and vegetable consumption, water intake, and feeling less stressed than the non-completers [25].

#### 2) Pre-determined interventions with some choice of activities

One study [26] had a fixed set of activities and some choice about which activities to participate in. In response to an identified need, a self-care plan and holistic learning programme on one hundred and three nurses’ health-promoting behaviours in intervention units over twelve months resulted in a significant difference pre- and post- intervention in nurses in intervention versus control units in overall Health Promoting Lifestyles Promotion (HPLP) II scores, and the sub-scales of stress management, nutrition, and spiritual growth [26].

#### 3) Choice and some adaptivity of the programme (supplementary activities)

Five interventions [23, 24, 27, 28, 32] offered an initial range of activities for the workforce to participate in, as well as providing supplementary activities during the implementation of the interventions.

Three of the five interventions involved “Workplace Champions” whose roles were delivery as well as gathering feedback and planning further activities [23, 24, 27]. In one intervention [23], the role of the workplace champion was to further refine and adapt the intervention activities delivered depending on identified need and context. Employee leadership and advisory boards were also created to develop site-specific strategies and approaches.

Three interventions [23, 28, 32] had an explicitly participatory approach both in the design and the delivery. Two [23, 28] included External Advisory Boards at each intervention site to engage the workforce and tailor activities to their needs and two had a strong emphasis on engagement of leadership and staff in the development and tailoring of intervention activities [28, 32]. The latter two contained activities designed to engage the whole worksite and develop relationships to support healthy behaviours, e.g. a directors’ team-building course, and activities for all staff (including leadership) to improve team coordination, communication and stress management [32].

Uptake of the intervention was not determined for any of the studies, probably as all offered a variety of activities as well as making some environmental changes; one study [24] reported that a third of all the employees participated in a competition organised as part of the intervention.

Four of the five studies [23, 24, 26, 27] reported an improvement in health and wellbeing behaviours (Table 3); increase in fruit and vegetable consumption (three-arm randomised controlled trial [23]); increased physical activity (before and after study with no control [27]; self-report, survey design, no control [24]); increase in healthy eating post-intervention [26]; and improved self-reported nutrition post-intervention in a controlled before and after study [26]. Two studies found no change in BMI [27, 28].

Three studies found improvements in employee mental health: improved job satisfaction (but no effect on mood or work perceived work performance [27]); more self-reports of staff satisfaction in post-intervention survey [24]; improvements in stress management and spiritual wellbeing post-intervention compared to controls [26].

One study [28] observed improved employee perception of worksite commitment to their health and wellbeing and that changes in perceptions of co-worker norms changed outcomes for participants: higher perception of co-worker normative healthy eating behaviours was associated with greater fruit and vegetable consumption and less fat consumption; and higher perception of co-worker normative physical-activity behaviours was associated with greater total physical activity. Perceived co-worker support also increased in the intervention arm in another study [23].

One study [28] observed a significant participation dose-response effect: When intervention exposure was used as the independent variable BMI decreased for each unit increase in intervention participation at 24 months (Table 3).

#### 4) Adaptive and responsive workplace programmes

Three large hospital studies [6, 29, 31] viewed the process of developing the intervention to be a part of creating a healthy workplace: a before and after study involving over three thousand employees from thirty seven regional hospital departments in Sweden [6]; a cluster-RCT in twenty four hospital departments in two hospitals in Japan [29]; and a cohort study comparing people exposed and not exposed to a workplace wellness champion intervention (self-reported) within a large academic medical centre [31]. The three interventions were adaptive and responsive to local needs and context from development and implementation, right through until the end of the study and beyond. This responsivity provides the opportunity for sustainability after the end of the study, and for processes involved in the intervention to become part of the workplace culture.

All three: aimed to improve the psychosocial work environment by utilising a participatory approach, asking each department or work area to identify the enablers and barriers to workplace wellbeing, and to set goals and identify areas for improvement; appointed key people or “champions” to support the interventions and to act as communicators within and across departments; and used feedback to develop activities responsive to local need (two [6, 29] fed back survey results to local staff and one [31] had workplace wellness champions (Table 3)design activities based on local feedback)

Two studies showed some evidence of a dose-response effect in which greater participation produced greater benefits. When a notice of staff cut-back was announced between baseline and follow-up staff in departments rated as highly active in improvement activities did not deteriorate during the follow-up period in work pressure, organizational climate and coping whereas staff in departments rated as less active did deteriorate [6]. Similarly, participants in a local work area who did versus did not participate in activities rated their overall health and wellness as significantly higher and significantly more of participating versus not participating in a local area agreed that their co-workers support one another in practicing a healthy lifestyle [31].

One [29] found varying levels of participation across the departments, with staff citing “realising that change was possible” and responding to identified needs as positive ways of improving the psychosocial work environment; concomitantly, the lack of time and common understanding, staff changes, and a feeling that activities were not responding to staff needs were given as reasons why the environment did not change. They observed no overall effect on mental health status, but significant increases in participatory management, co-worker support, and job control versus control.

### Recommendations 3, 4, & 5: Engagement, involvement and upskilling of management and board-level staff

Three of the five recommendations involve the engagement and support of management and board-level staff in intervention activities, including strong visible leadership, support for the health and wellbeing of staff, and the targeting of resources on improving management capability and capacity to deliver this increased visible leadership and support. Despite this emphasis in the recommendations these activities were notably lacking in seven interventions reviewed. Promisingly, five focused considerable resource on engagement, involvement and upskilling of management staff [6, 28, 29, 31, 32]. Two significantly improving mental health and wellbeing of healthcare staff [29, 31], one reducing deterioration in mental health measures for people with high versus low participation rates [6], one significantly improving physical health (BMI) and health behaviours [28]. The fifth found no significant effect on mental health and wellbeing [32].

Four interventions involved extensive management involvement as champions [28, 29, 31, 32]. Sun and colleagues [32] Engaging higher management staff in local staff activities and creating opportunities for increased communication, group solidarity and group coordination, alongside intervention activities to improve leadership and management and communication skills of higher levels of management (e.g. directors of intervention centres were engaged and educated about the importance of activities in groups for staff, and then were involved in implementing and taking part in the group activities, having the responsibility to plan, coordinate, and monitor the group’s activities and to convey a vision, inspiring team collaboration) resulted in no significant impact of their intervention on workplace social capital, the measures of which included items on vertical and horizontal trust and communication. In another intervention [31], following efforts to involve management, supervisors and HR in supporting the workplace wellness champions to deliver and implement their locally adapted intervention, twenty-three percent of people engaging with the intervention reported an improved work atmosphere, and significantly more of those participating strongly agreed or agreed that the organisation provides a supportive environment to live a healthy lifestyle compared to those not familiar with the wellness champions. Enthusiastic“buy in” at the upper level of administration and visible strong leadership support, when it improves cooperation by other staff to implement changes [28], produced a significant association between perception of stronger organisational commitment to employee health and a reduction in BMI. When local leadership staff were directly supported to develop their capability and capacity to improve staff health and wellbeing (Table 3) [29], the intervention group showed a statistically significant increase in the psychosocial work environment questionnaire sub-scale of ‘participatory management’, along with self-reports of improved work environment in the process evaluation; however, there was no significant difference in the intervention groups’ scores on the depression scale pre- to post- intervention.

When there was a focus on management visibility and involvement in the feeding back of local results and the active implementation of changes related to locally raised needs, and improving vertical communication between managers and staff, staff in departments that actively participated showed significantly less deterioration in perceived organisational climate compared with staff in departments with low participation [6].

## Discussion

This systematic review identified eleven studies of workplace health promotion interventions which sought to enhance the health and wellbeing of healthcare staff by using a whole-system approach to interventions. The low number of identified studies highlights that the impact of whole-system healthy workplace interventions for healthcare workers, as recommended by Boorman [17] is under-researched, and we feel this gap is important for future research to address.

Although the studies were of mixed (mid to low) quality and the intervention designs varied considerably, the reported results suggest that interventions taking a whole-system approach can improve physical and mental staff health and wellbeing and promote healthier behaviours.

Interventions that incorporate at least one of the five whole-system recommendations for improving healthcare worker health and wellbeing [12] resulted in improvements in physical and/or mental health and promoted healthier behaviours in healthcare staff. However, one study [32] incorporated all five of the recommendations in their workplace social capital intervention and did not find any significant change in measures of mental health (workplace social capital measure, see Table 2). It is not possible to draw conclusions regarding the specificity of the interventions as they varied widely in terms of their context, development, design, and implementation, but it is interesting to note that there seems to be no relation between the greater number of recommendations incorporated in interventions and the effectiveness of the study. However, heterogeneity in outcome measures makes this a tentative comparison.

Four studies of interventions offering choice of a range of activities to participate in [6, 23, 28, 31] individually offer some evidence that the greater the level of participation, the greater the individual benefit: greater participation improved: resilience to organisational change [6]; self-rated overall health and wellness [31]; BMI [28]; and fruit and vegetable consumption [23]. The latter study also found enhanced effect of a worksite intervention when there was family participation suggesting that widening activities beyond the workplace to include family and friends may further enhance engagement and improvements to wellbeing.

The suggestion of potential individual “dose-response effect” in these four studies (i.e. more benefit derived from more participation [6, 23, 28, 31] has several implications: firstly, it suggests that attention needs to be given to creating intervention activities that healthcare staff want to engage in and offering a selection of a range of activities, some team- and some individual-based, for participants to choose between. Interestingly, some workplace interventions [28] had the least take-up of team-based activities, whereas others [24] found this the most participative aspect of the intervention.

The four studies that reported findings from their process evaluations [23, 25, 28, 29] all suggested that time was one of the greatest barriers to employee participation in workplace health and wellbeing interventions. Understanding the barriers to participation (such as time, resources, and poor communication about activities) should be part of the process of evaluating any workplace intervention, and having an intervention able to adapt to allow different times and ways of participating should be beneficial.

Although it is hard to make any meaningful comparisons regarding effectiveness, the studies which assessed intervention activity participation [23, 25] suggest that the interventions in which staff were involved from the beginning in determining the activities had greater participation. There was lower participation in interventions with more pre-determined activities, even when there was an opt-in process for staff and hence potentially had a more motivated workforce participating. One study [25] invited 400 of 2900 staff to participate in a 12 week intervention and had 61% participation, compared with the 81% participation in the workplace intervention implemented by another study [23] where employees were involved in the development and implementation of their workplace intervention.

### Implications for policy/practice

The Boorman Review [17] called for healthcare workplaces that: support local staff needs; have staff engagement at all levels; have strong visible leadership and support at senior management and board level on health and wellbeing; have a focus on management capability and capacity to improve staff health and wellbeing. Our systematic review shows that interventions incorporating these whole-system approaches can improve healthcare staff health and wellbeing and increase health behaviours.

Only five of the eleven studies focussed on management capability and capacity. There was some evidence from subjective author reports that this focus resulted in enthusiastic engagement from leadership [28, 29, 32]. Of these five interventions, four involved management-level staff as healthy workplace champions. It is interesting to note that the findings of these four studies all involved perceptions of improved workplace culture or atmosphere in participants: improved work atmosphere and environment, more supportive environment to live a healthy lifestyle, more ‘participatory management’, less deterioration in perceived organisational climate, and stronger organisational commitment to employee health (the latter of which was significantly associated with reductions in objectively measured BMI). However, the fifth study found no significant impact of their intervention on measures of vertical trust and communication. These findings provide some evidence that interventions including efforts to engage and involve management staff, such as in the feedback of local results of health and wellbeing surveys and involvement in discussions with local staff of how they would like to address the, in being workplace champions themselves, to make their leadership on health and wellbeing more visible, and to provide training on skills to support the health and wellbeing of their staff, can impact the perception by those staff that management are on their side and that they work in a place with a positive workplace environment.

The finding of potential “dose-response” effects in the four studies that report participation rates suggest that participation and engagement are important in designing and implementing healthy workplace interventions: A flexible intervention with continuous employee involvement and an ongoing evaluation to highlight facilitators and barriers to participation has greater potential to positively affect and sustain health and wellbeing for the healthcare workforce and thus to improve staff health and wellbeing.

### Strengths and limitations

The review was conducted according to the principles published by the NHS Centre for Reviews and Dissemination (CRD) and is reported according to PRISMA guidelines (S3 Fig). The review was comprehensive, searching across electronic and grey literature sources to identify studies. There were no language or date restrictions in the searches.

Due to the nature of the topic under consideration, the inclusion criteria in this review were open to a degree of subjective interpretation. For this reason we took all reasonable steps to ensure that eligibility criteria were applied consistently across all identified articles by 1) piloting the criteria on a subset of papers, 2) having two reviewers independently assess the eligibility of all articles with discussion of all disagreements, and 3) involvement of a third reviewer to resolve disagreements where necessary.

Comparison across the approaches utilised to improve health and wellbeing of healthcare professionals was challenging due to the lack of detail provided regarding the specific nature of the components and the mechanisms making up the interventions.

Variable methodological quality, mostly related to the outcome measures used, which were in general low in reliability, variable in validity, and heterogeneous, along with the nature of the study designs prevented any conclusions related to the effect on health and wellbeing outcomes of incorporating more versus less of the five whole-system recommendations being made. Nor were we able to compare the effectiveness of different patterns of whole-system recommendation implementation.

### Recommendations for future research

Despite extensive and systematic searching of the literature, we were only able to identify eleven studies that met our inclusion criteria. The low number of identified studies highlights that there is currently limited evidence regarding the effectiveness of whole-system approaches to enable staff health and wellbeing for healthcare professionals in healthcare settings, as recommended by Boorman [17]. Ten out of eleven included studies provide evidence that whole-system approaches to healthcare workplace health interventions that include at least one of the five whole-system recommendations [17] improve physical and/or mental health and promote positive health behaviours in healthcare staff, suggesting this is an area of potentially fruitful inquiry.

The methodological quality of the studies was mostly low, with only five out of eleven studies included being rated as “medium” quality. This systematic review clearly identifies a need for good quality primary research using similar and validated outcome measures to evaluate whole-system approaches to health and wellbeing interventions in healthcare worker populations. Comparative studies of the effectiveness of individual-focused versus whole-system-focused approaches would clarify their relative effectiveness and cost-effectiveness. Long-term follow-up is necessary to evaluate the sustainability of observed change. More systematic reporting would allow more definitive conclusions about how the conditions for sustainable healthy workplaces for healthcare workers can be created.

The low number of studies and heterogeneous intervention designs and outcome measures used in those studies, makes it challenging to pin down whether and in what way whole-system approaches improve healthcare worker health and wellbeing. Realist reviews of the literature, in which context-mechanism-outcome configurations rather than whole interventions are the unit of analysis, would be of use in establishing what it is about whole-system interventions that works to improve health and wellbeing for healthcare workers, who for, under what circumstances, and in what way.

## Conclusion

This systematic review identified 11 studies which incorporate at least one of the Boorman recommendations and provides evidence that whole-system healthy workplace interventions can improve health and wellbeing and promote healthier behaviours in healthcare staff.

## Supporting information

S1 Fig Search strategy. (DOC)

S2 Fig Data extraction form. (DOCX)

S3 Fig PRISMA 2009 checklist. (DOC)
