# Supplementary material for: Whole-system approaches to improving the health and wellbeing of healthcare workers: A systematic review
Source: PLoS One. 2017 Dec 4;12(12):e0188418. doi: 10.1371/journal.pone.0188418 (PMC5714334; doi:10.1371/journal.pone.0188418)
Supplement: S1 Fig — (DOC) [file pone.0188418.s001.doc]

Database: Ovid MEDLINE(R) Search Strategy:

--------------------------------------------------------------------------------

1 exp Health Personnel/

2 exp Workplace/

3 exp Intervention Studies/

4 exp Health Promotion/

5 exp Healthy People Programs/

6 exp Health Knowledge, Attitudes, Practice/ or exp Health Literacy/

7 exp "Quality of Life"/

8 exp Personal Satisfaction/

9 exp Smoking/ or exp Smoking Cessation/

10 exp Eating/

11 exp Food Habits/

12 exp Alcoholic Beverages/

13 exp Alcohol Drinking/

14 exp Drinking Behavior/

15 exp Life Style/

16 exp Job Satisfaction/

17 exp Health Status/

18 exp Physical Fitness/

19 exp Health Behavior/

20 (staff or personnel or employe* or profession* or workplace* or worksite or practioner or nurse*).tw.

21 (intervent* or promot* or initiativ* or program* or scheme*).tw.

22 (health or healthy or wellness or wellbeing or exercise* or food* or diet* or alcohol* or smok* or leisure or fit* or stress* or depress* or activ*).tw.

23 ((intervent* or promot* or initiativ* or program* or scheme*) adj (health or healthy or wellness or wellbeing or exercise* or food* or diet* or alcohol* or smok* or leisure or fit* or stress* or depress* or activ*)).tw.

24 ((staff or personnel or employe* or profession* or workplace* or worksite or practioner or nurse*) adj5 ((intervent* or promot* or initiativ* or program* or scheme*) adj (health or healthy or wellness or wellbeing or exercise* or food* or diet* or alcohol* or smok* or leisure or fit* or stress* or depress* or activ*))).tw.

25 1 or 2

26 3 or 4 or 5 or 6

27 25 and 26

28 7 or 8 or 9 or 10 or 11 or 12 or 13 or 14 or 15 or 16 or 17 or 18 or 19

29 27 and 28

30 24 or 29
