# Supplementary material for: Whole-system approaches to improving the health and wellbeing of healthcare workers: A systematic review
Source: PLoS One. 2017 Dec 4;12(12):e0188418. doi: 10.1371/journal.pone.0188418 (PMC5714334; doi:10.1371/journal.pone.0188418)
Supplement: S2 Fig — (DOCX) [file pone.0188418.s002.docx]

**Data extraction form for healthy workplaces review**

BIBLIOGRAPHIC DETAILS

Reference no:

Insert

First author:

Insert

Title:

Insert

Year:

Insert

*Citation:*

Insert

**STUDY DETAILS**

Country:

Insert

*Is the study in a hospital setting?*

Insert

*Is the study experimental or observational?*

Experimental

*Type of Study (please tick):*

*☐ RCT*

*☐ Quasi-randomised trial*

*☐ Controlled before and after study*

*☐ Before and after study (no control group)*

*☐ Cohort study*

*☐ Other, please specify:*

*Ethical approval:*

Insert

Funding source:

Insert

Notes:

Insert

**INTERVENTION**

What was the stated aim of the intervention?

Insert

What was the intervention?

Insert

Was it individual or group based?

Insert

Who delivered the intervention?

Insert

Did the deliverers need any training or qualifications?

Insert

How many sessions were there?

Insert

How long did each session last?

Insert

Over what period of time was the intervention delivered?

Insert

Where was the intervention delivered?

Insert

Did it have an educational component?

Insert

(If control/comparator group) what happened to the control group?:

Insert

Notes:

Insert

Is the intervention described in sufficient detail for it to be replicated elsewhere?

Insert

**PARTICIPANTS**

What type of health professionals were participants?

Insert

How were participants recruited?

Insert

Total number invited to participate:

Insert

Agreed to participate:

Insert

Total participated:

Insert

Number of participants in intervention condition:

Insert

Number of participants in control condition:

Insert

Total number of participants excluded/lost at follow up:

Insert

T1 Number excluded/lost at first follow-up from intervention ( ) or from control ( )

T2 Number excluded/lost at second follow-up from intervention ( ) or from control ( )

**PARTICIPANT CHARACTERISICS**

*Mean (SD) Control (if applicable): Intervention:*

*N*

*Age yrs*

*Male*

*Female*

*Other characteristics provided:*

Insert

OUTCOME MEASURES

Does the study have measures relating to any of the following outcomes? (it must have at least one of the below, please tick):

☐ psychological wellbeing/stress

☐ diet

☐ physical activity

☐ alcohol consumption

☐ smoking

Primary outcome measure/(s) used (please specify whether or not each measure is validated):

Insert

Secondary measures:

Insert

Method of assessing outcomes:

Insert

Outcome measures at what time points?

Insert

Notes:

Insert

Who carried out the data collection?

Insert

RESULTS

Please use the blank table or space to enter the results.

**GENERAL COMMENTS:**

*Data extraction performed by: Date:*

Data extraction checked by: Date:
